# Fabrication of Highly Oriented Cylindrical Polyacrylonitrile, Poly(lactide-*co*-glycolide), Polycaprolactone and Poly(vinyl acetate) Nanofibers for Vascular Graft Applications

**DOI:** 10.3390/polym13132075

**Published:** 2021-06-24

**Authors:** Sairish Malik, Subramanian Sundarrajan, Tanveer Hussain, Ahsan Nazir, Seeram Ramakrishna

**Affiliations:** 1 Electrospun Materials & Polymeric Membranes Research Group (EMPMRG), National Textile University, Sheikhupura Road, Faisalabad 37610, Pakistan; malik.sairish@gmail.com (S.M.); Hussain.tanveer@gmail.com (T.H.); ahsan.nazirr@gmail.com (A.N.); 2 Department of Mechanical Engineering, National University of Singapore, 9 Engineering Drive 1, Singapore 117576, Singapore; seeram@nus.edu.sg

**Keywords:** orientation, cylindrical, collectors, vascular grafts, cardiovascular diseases, mechanical strength

## Abstract

Small-diameter vascular grafts fabricated from synthetic polymers have found limited applications so far in vascular surgeries, owing to their poor mechanical properties. In this study, cylindrical nanofibrous structures of highly oriented nanofibers made from polyacrylonitrile, poly (lactide-*co*-glycolide) (PLGA), polycaprolactone (PCL) and poly(vinyl acetate) (PVAc) were investigated. Cylindrical collectors with alternate conductive and non-conductive segments were used to obtain highly oriented nanofibrous structures at the same time with better mechanical properties. The surface morphology (orientation), mechanical properties and suture retention of the nanofibrous structures were characterized using SEM, mechanical tester and universal testing machine, respectively. The PLGA nanofibrous cylindrical structure exhibited excellent properties (tensile strength of 9.1 ± 0.6 MPa, suture retention strength of 27N and burst pressure of 350 ± 50 mmHg) when compared to other polymers. Moreover, the PLGA grafts showed good porosity and elongation values, that could be potentially used for vascular graft applications. The combination of PLGA nanofibers with extracellular vesicles (EVs) will be explored as a potential vascular graft in future.

## 1. Introduction

Cardio vascular diseases (CVD) are increasing day by day and becoming one of the primary causes of annual deaths around the world [1]. Coronary artery (CA) disease is one of the most noticeable forms of cardiovascular diseases [2], whereas the main cause of CVD is the blocking of blood vessels, limiting blood supply to vital organs [3]. Current treatment choices depend on surgical revascularization by implantation of a graft to bypass the blockage. [4] Surgeons usually use the saphenous vein during CA surgery; though, removal of the vein becomes a cause of major indisposition at donor site [5]. Furthermore, limited availability of autologous grafts also limits their application. Thus, a feasible option for the bypass of blocked small diameter blood vessels is highly desirable. Tissue engineered vascular grafts have made outstanding progress [6,7]. Tissue engineering (TE) has been used for surgery of tissues and organs [8]. In TE, a three dimensional (3D) scaffold has been used for repairing the defected/damaged blood vessels, which mimic the structure of extracellular matrix (ECM) of blood vessels [9]. Even though, various materials have been explored to design the nanoscale features of scaffolds, but polymeric nanofibers have been reported as one of the best options to mimic the native structure of ECM [10,11]. Even though different techniques can be used to fabricate nanofibers, viz phase separation [12,13], drawing [14], template synthesis [15,16,17], self-assembly [18,19] and electrospinning [20,21], but electrospinning has gained a lot of importance, due to its ability to fabricate scaffolds that mimic the well-ordered architecture of native ECM of tissues [22]. It is a smart choice, being simple, cost effective and environmentally friendly [23]. Furthermore, it can fabricate nanowebs in specific structures with controlled orientation and diameter [24,25]. 

In recent years, nanofibers have been used for various biomedical applications [26,27]. Even though randomly collected nanofibers can be used in various fields of TE, but in some applications such as vascular grafts, it is essential that the nanofibers should also be axially oriented [28], as the orientation of the nanofibers influences the mechanical properties, cell growth and relevant cell functions [29]. Even though a wide range of electrospinning setups have been developed to acquire oriented nanofibers, rotating drum/disk collectors are some of the most widely used to acquire oriented fibers. The degree of alignment within the nanofibers can be controlled by varying the speed of the rotating drum/disk collector. At low speed, randomly oriented nanofibers are collected. However, with increasing the speed, the degree of fiber orientation improves. 

Researchers have investigated use of various types of collectors for nanofibers orientation in a specific direction, yet limited work has been accomplished in the field of orienting the nanofibers in cylindrical shape for vascular grafts. In one of the studies, round collectors with conductive parts separated by insulating gaps were used to acquire orientated nanofibers in the direction of electric field lines in the gap between the two conductive parts. However, this was not successful as the insulating gap was not high enough to achieve the orientation. As the gap increased, the influence of electric field lines on the nanofibers was reduced and control on fiber orientation was also decreased [30,31]. In another study, Teflon rod was used between the metallic plates, but nanofibers orientation was poor, due to high flow rate and low rotational speed of collectors. Later, it was found that significantly high rotational speed was necessary to initiate the process of fiber stretching and thereby fiber orientation. Various researchers have also worked on the orientation of nanofibers, by using a rotating disk of having an insulating tube on the collector [32,33]. However, when the nanofibers layers were increased on the rotating collector, the influence of electrostatic forces was reduced and formation of fiber orientation was affected [34]. Even though, variety of innovative techniques have been introduced to acquire the nanofibers oriented in a specific direction, but it is challenging not only to form better oriented fibers, but also to explore them for vascular graft applications due to their insufficient mechanical strength. This work is aimed to overcome the previous drawbacks by using novel collectors of having alternate conductive and non-conductive segments. We have used a multi-segmented stand- alone collector, in which the lengths of segments have been optimized after several experiments [35,36] to achieve highly oriented nanofibers from four different polymers viz polyacrylonitrile (PAN), poly(lactide-co-glycolide) (PLGA), polycaprolactone (PCL) and poly(vinyl acetate) (PVAc). PAN was selected due to its low cost, non-toxicity, high strength and high modulus of elasticity, and has been used in many medical applications [37,38,39,40,41]. PLGA has good mechanical properties and biodegradability [42], PCL has good biomechanical properties and cell compatibility [43], PVAc also has good biocompatibility [44] and has been used in vascular grafts applications. The overall aim of the study was to find the polymer with best properties of mechanical strength, burst pressure and suture retention strength, that could be used in future for vascular grafts applications.

## 2. Materials and Methods

Four different polymers, viz., PAN (*M*_w_: 150,000 g/mol), PLGA (*M*_w_: 60,000 g/mol), PCL (*M*_w_: 80,000 g/mol) and PVA (*M*_w_: 500,000 g/mol) were purchased from Sigma Aldrich (St. Louis, MO, USA). Solvents such as *N*,*N*-dimethyl formamide and 1, 1, 1, 3, 3, 3-Hexafluoro-2-propanol (≥99%) were supplied by Sigma Aldrich (St. Louis, MO, USA). All the polymer solutions were prepared separately by adding the required amount of polymer and the solvent into a flask with a magnetic stirring for 24 h.

### 2.1. Electrospinning

**Figure 1:**
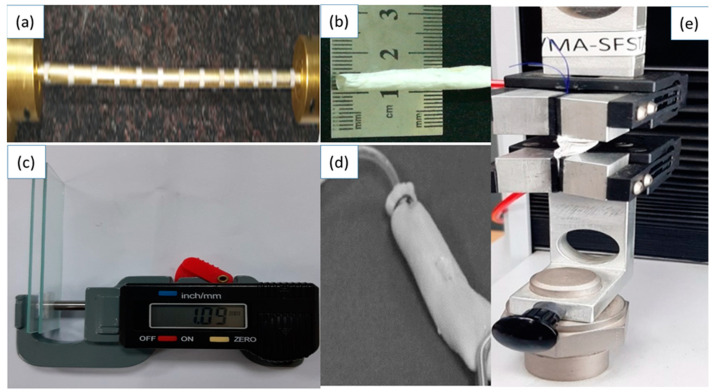
Fabrication and testing of vascular grafts. (**a**) Cylindrical collectors with conductive and non-conductive segments, (**b**) electrospun cylindrical vascular graft, (**c**) Thickness measurement, (**d**) bursting pressure measurement, (**e**) measurement of tensile strength and suture retention strength.

The electrospinning of each polymer solution was accomplished separately using Fluidnatek electrospinning set-up, and cylindrical collectors (300 mm length × 5 mm dia.), manufactured in-house, comprising two parts: conductive part (CP) (brass) and non-conductive part (NCP) (Teflon) as depicted in Figure 1. The electrospinning parameters were kept the same for all the polymer solutions, i.e., polymer concentration 11 wt %, electrospinning needle gauge size of 21, voltage 12 KV, needle-to-collector distance 14 cm, solution flow rate 150 μL/h, and collector rotational speed of 100 rpm. Groove depth of the collector was kept at 2 mm. All of the parameters were selected from the previously reported procedure.

Axially aligned scaffolds were collected on the rotating collectors. When two conducting parts of the collector, separated by non-conducting parts, are used, the fiber jet will deposit on the non-conducting part in between the conducting parts and a horizontally aligned array of nanofibers will be produced. The electric field lines in the non-conducting part near the conducting part are drawn towards the conducting part. The fiber jet stretches across the non-conducting part as the field lines are attracted towards the conducting part. This in turn orients the nanofibers on the non-conducting part of the collector. Due to presence of charge on the nanofibers, mutual repulsion between the deposited nanofibers will enhance the parallel and relatively even distribution of nanofibers. This process is repeated in all the non-conducting parts surrounded by the conducting parts and thus an array of longitudinally oriented nanofibers could be collected on the entire collector. After spinning, the electrospun vascular grafts were removed from the collector and stored in a desiccator for further testing and characterizations. Preparation and testing of scaffolds are shown in Figure 1.

### 2.2. Scanning Electron Microscopy

**Figure 2:**
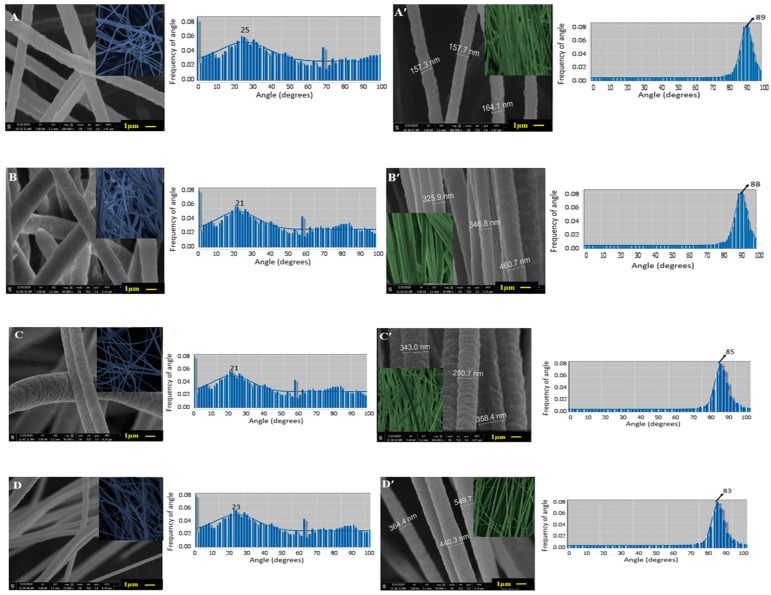
SEM micrographs and corresponding frequency distribution of orientation angles of nanofibrous structures: PAN (**A**: not aligned; **A’**: aligned); PLGA (**B**: not aligned; **B’**: aligned); PCA (**C**: not aligned; **C’**: aligned); PLA (**D**: not aligned; **D’**: aligned).

Samples were coated with a thin layer of gold to make nanofibers conductive. Then a Field emission scanning electron microscope was used to determine the surface morphology and diameter of the nanofibers at an accelerating voltage of 100 KV and 50 KV. The Java image processing software [Image J 1.52] was used to measure the diameter of the nanofibers (n = 20). The orientation of nanofibers was quantified by using orientation J, a plugin for image J 1.52 software. SEM micrographs of the region of interest (ROI) were used to find out the orientation values. Samples having high orientation values (aligned nanofibers) and low orientation values (not-aligned nanofibers) (Figure 2) were selected for further testing. 

### 2.3. Thickness 

Thickness of the electrospun graft samples was measured using a digital caliper on glass slides (Figure 1c). Dimension of each glass slide was 3ʺ × 1ʺ × 1ʺ. Samples were placed between the slides, and thickness was measured according to D 6988-13 standard test method. Each sample was measured from the center, on the right and on the left. The average of these three measurements taken from millimeters was recorded as the thickness of samples. For porosity measurement, the samples were accurately cut about 1cm^2^ size and weighted to determine apparent density. The porosity of the nanofibers was calculated using the formula given below:

(1) $$\left[1-\frac{\left(Apparent\;density\;of\;fiberous\;strip\right)}{Bulk\;density\;of\;Polymer}\;\right]\times 100$$

The bulk densities of PAN, PCL, PVA and PLGA were taken as 0.36 g/cm^3^, 1.14 g/cm^3^, 1.19 g/cm^3^ and 0.75 g/cm^3^, respectively.

### 2.4. Mechanical Properties

Mechanical properties of longitudinally aligned vascular grafts were determined by using a universal testing machine (UTM) according to a standard test method (ASTM D 882-01). The samples were cut into 50 mm length and 5 mm width. Load deformation values were recorded at 10 mm/min speed and 20 mm gauge length, using 100 N load cell at a constant speed of 0.1 mm/s.

### 2.5. Suture Retention Test

Suture retention test was performed on the universal testing machine (UTM) with a load cell of 100 N. For determining suture retention strength, the vascular grafts were cut into strips having dimension of 40 mm × 5 mm. A Prolene suture 2-0 was passed through the strip and surgical knot was achieved 2 mm from the edge of the sample. At one side of the machine, an electrospun strip was clamped and on the other side, the suture was clamped. Then for testing, suture was pulled at a rate of 50 mm/min until it tore out from the strip. The force required to pull the suture was recorded as a suture retention strength.

### 2.6. Burst Pressure

For burst pressure measurement, 5 mm diameter cylindrical grafts were taken (n = 5). The cylindrical graft was clamped on both sides in a glass chamber at 37 °C. The flow rate was adjusted in the range of 3–6 mL/min while the pressure was adjusted between 18–75 mmHg. In order to determine the blood pressure, the initial pressure was increased, keeping the flow rate the same. For the determination of burst pressure, the pressure was increased until the sample showed complete burst. The highest pressure measured before rupture was considered as burst strength.

## 3. Results and Discussions

### 3.1. Surface Morphology and Fiber Orientation and Fiber Diameter

SEM images of not-aligned and aligned scaffolds are shown in Figure 2.

No signs of beads and entanglement of nanofiber were observed in the SEM images. The orientation values of aligned scaffolds were found to be PAN: 89 ± 1.58 degree, PLGA: 88 ± 1.67 degree, PCL: 85 ± 1.58 degree, and PVA: 83 ± 1.3 degree. The orientation values of not-aligned scaffolds were found to be PAN: 25 ± 1.58 degree, PLGA: 24 ± 1.56 degree, PCL: 21 ± 1.26 degree and PVA: 23 ± 1.14 degree. Directional analysis confirmed the alignment of nanofibers in the scaffolds. For not-aligned scaffolds, no proper fiber direction was predominant, while in the aligned scaffolds, a prominent direction was observed and a peak was formed. In the cases of not- aligned scaffolds, the histogram is wider while in the cases of aligned scaffolds, the histogram is narrower, which shows the orientation of fibers is in the same single direction. The average diameters of the nanofibers in aligned scaffolds were found to be, PAN: 159 ± 7 nm, PLGA: 378 ± 20 nm, PCL: 327 ± 25 nm and PVA: 451 ± 40 nm. The average diameters of nanofibers in not-aligned scaffolds were found to be PAN: 162 ± 10 nm, PLGA: 379 ± 20 nm, PCL: 329 ± 30 nm and PVA: 452 ± 30 nm. When we compared the morphology of these nanofibers, it was found that both PLGA and PCL have comparatively rougher exterior morphology (Figure 2A,A′,C,C′ than other samples (Figure 2B,B′ and Figure 2D,D′, and the latter reveals smooth morphology. Figure 2B shows the histograms of diameter distributions of nanofibers at different polymer concentration, conductive segment length (CSL) and non- conductive segment length (NSL). It can be noted that distribution of nanofibers diameters varies for aligned and not-aligned nanofibers.

### 3.2. Porosity

The porosity of scaffolds was calculated. It was observed that the porosities of non- aligned scaffolds were 93%, 95%, 92% and 94% for PAN, PLGA, PCL and PVA, respectively, while the porosities of aligned scaffolds were 85%, 87%, 78% and 86%, respectively. As shown in Figure 2, small and narrow pores are visible on aligned scaffolds, whereas large pores are visible in the case of not- aligned nanofibers. The aligned scaffolds structures give a denser structure and lower porosity than not-aligned scaffolds [45].

### 3.3. Tensile Strength

**Figure 3:**
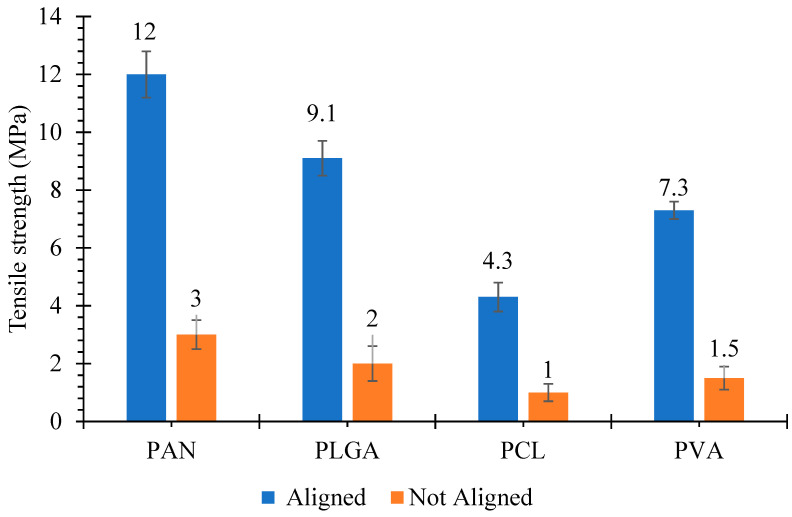
Tensile strength of cylindrical nanofibrous structures, with ‘aligned’ and ‘not aligned’ fibers.

The tensile strength of the scaffolds is shown in Figure 3. For the random electrospun grafts, two clamps of the tensile machine may grip both ends of individual electrospun fibers. Therefore, the stress by the tensile machine is exerted on the individual fiber during the test and then weaker fibers start to break one by one. On the other hand, for the aligned electrospun grafts, the clamp of the tensile machine may grab only one end of the fibers and these fibers are connected with other fibers. Therefore, when the machine stretches the electrospun grafts, the stress is exerted on the fiber networks, rather than individual fibers. During the test, it seems that rearrangement of the fiber networks happens, resulting in the elongation of electrospun grafts. Hence more force is needed to break the grafts.

It was revealed that tensile strength values were varied for both aligned and not-aligned grafts. In the cases of aligned scaffolds, the PAN scaffolds had the highest mechanical strength of 12 ± 0.8 MPa, while the PCL scaffold had the lowest mechanical strength of 4.3 ± 0.5 MPa. Tensile strength values of PLGA and PVA were 9.1 ± 0.6 and 7.3 ± 0.3 MPa, respectively. Tensile strength values for not-aligned scaffolds were 3 ± 0.21, 2 ± 0.15, 1 ± 0.16 and 1.5 ± 0.14 MPa for PAN, PLGA, PCL and PVA, respectively. It was observed that tensile strength values for aligned scaffolds were greater than that of not-aligned scaffolds. The increase in tensile strength of aligned scaffolds is due to the dense structure of aligned scaffolds. Moreover during tensile loading, fibers that are aligned in the direction of loading experience the stretching force hence show resistance against force [45]. Similar observation of aligned scaffolds with enhanced mechanical properties than not-aligned scaffolds was already reported by Neethu Mohan et al. [46].

**Figure 4:**
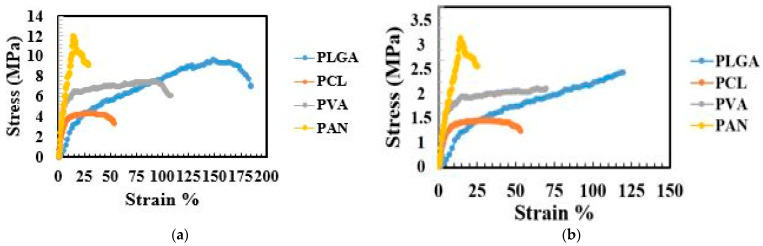
Stress-strain curves of cylindrical nanofibrous structures, (**a**) with ‘aligned’ fibers, (**b**) with ‘not aligned’ fibers.

A comparison of stress strain behavior for aligned scaffolds and not-aligned scaffolds is displayed in Figure 4.

PLGA showed the highest strain (elongation) of 183% at breaking point with a tensile strength of 9.1 MPa. Similarly, the elongation values at break of PAN, PCL and PVA were 29%, 53% and 107%, respectively. While the tensile strength values for not-aligned scaffolds were comparatively lower and elongation at break values were also low, i.e., 120%, 50%, 70% and 25% for PLGA, PVA, PCL and PAN, respectively. It has been reported that femoral arteries show ultimate stress of 1–2 MPa and elongation at break values of 60–80% [47,48]. In the cases of PLGA and PVA scaffolds, they had good mechanical properties, which can satisfy the mechanical properties of artificial vascular grafts. On the other hand, PAN and PCL showed good stress values, but elongation values at break were low. 

### 3.4. Suture Retention Strength

**Figure 5:**
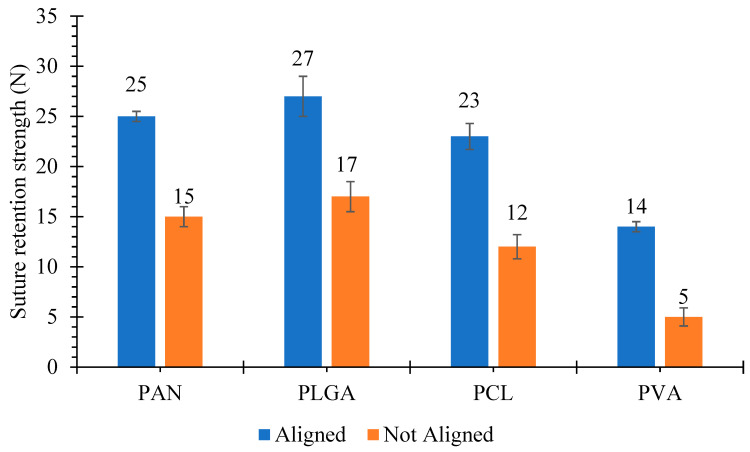
Suture retention strength of cylindrical nanofibrous structures, with ‘aligned’ and ‘not aligned’ fibers.

Suture retention strength values for aligned and not-aligned scaffolds are displayed in Figure 5.

In each graft type, five samples were analyzed and mean values of suture retention strength were recorded. In the cases of aligned scaffolds, PLGA had the highest suture retention strength with a value of 27 ± 2 N, whereas PVA had the lowest suture retention strength of 14 ± 0.5 N. Suture retention strength values of aligned PCL and PAN were 23 ± 1.3 N and 25 ± 0.5 N, respectively. The suture retention strength values for not- aligned scaffolds such as PAN, PLGA, PCL and PVA were 15 ± 1 N, 17 ± 1.5 N, 12 ± 1.2 N and 5 ± 0.9 N, respectively. These suture retention values were comparatively higher than the internal mammary arteries and human saphenous veins [49]. The obtained results were significantly higher than the study conducted by Zahang et al. [50]. The suture retention strength of the grafts were 3.30 ± 1.35 N and 3.79 ± 0.67 N. Both of them were greater than those of the human internal mammary artery (1.40 ± 0.01 N) and the human saphenous vein (1.81 ± 0.02 N). 

Suture retention strength is important to ensure that graft can withstand physiological pressure during and after implantation. It is well known that suture retention is highly related to orientation of nanofibers in the scaffolds. In the case of not-aligned scaffolds, the randomly distributed nanofibers make a network, which can hinder the passage of structure, but all the nanofibers cannot be obstructed, hence suture retention value is less [51,52].

### 3.5. Burst Pressure

**Figure 6:**
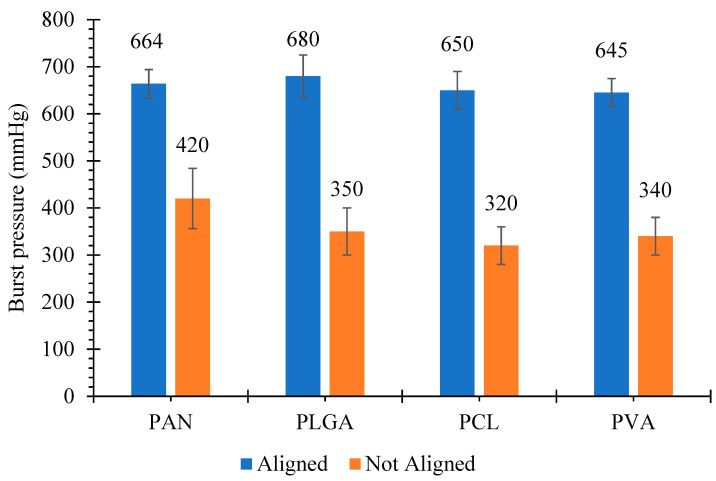
Burst pressure of cylindrical nanofibrous structures, with ‘aligned’ and ‘not aligned’ fibers.

Testing of burst pressure is a vital parameter to confirm that a vascular graft can endure the blood pressure after implantation. The normal pressure in the arterial circulation is 100 mmHg, and during standing it further increases in the case of leg arteries. Due to the influence of hydrostatic pressure, the pressure can reach up to 250 mmHg [53]. The mechanical strength and burst pressure required for arterial implantation are not yet defined properly, but burst pressures of 600–700 mmHg has been suggested in porcine models [54]. In our study, not-aligned nanofibrous structures made from PAN, PLGA, PCL and PVA showed burst pressure of 420 ± 64, 350 ± 50 320 ± 40 and 340 ± 40 mmHg, respectively, while the burst pressures for structure with aligned fibers were 664 ± 30 mmHg, 680 ± 35 mmHg, 650 ± 40 mmHg and 645 ± 30 mmHg, respectively, as shown in Figure 6.

These values are comparable to the values reported in the literature [55]. Notably, all the grafts showed adequate strength (2–6 times) to physiological pressure of 120 mmHg for implantation. Many researchers reported that these burst pressure values are safer for in vivo implantation [56,57]. The ISO guidelines 7198 contain information about evaluation of burst pressure, but the significant amount of pressure required for safer implantation is not given in the guidelines, hence the optimum burst pressure for artificial vascular grafts still needs to be focused. 

Even though vascular grafts are successfully developed in the literature, but there are some constraints in clinical translations, such as low cell survival rates and poor proliferation. We will be developing the grafts which are cell-free in future, which will be achieved by the combination of extracellular vesicles (EVs) and nanofibrous scaffolds. EVs are directly released from the cell membrane from various types of cell and involved in immune signaling, angiogenesis, stress response, proliferation and cell differentiation [58]. 

### 3.6. Statistical Analysis

All the data presented are expressed as mean ± standard deviation and were analyzed using Analysis of variance (ANOVA) for the effect of different polymers and alignment of nanofibers on different response variables is given in Table 1.

In ANOVA Table 1, P-values below 0.05 indicate that the effect of the parameter is statistically significant, whereas a higher value of SS (Sum of square deviation) of a parameter indicates its higher influence on the response variable as compared to the other parameter. According to Table 1(a),(c)–(e), both the type of polymer as well as the orientation had significant effect on fiber orientation, tensile strength, suture retention strength and burst pressure. Higher values of SS for alignment as compared to polymer, in Table 1(c)–(e), indicates that alignment has higher influence on tensile strength, suture retention strength and burst pressure as compared to the type of polymer. Table 1(b) shows that *p*-value for alignment is not significant, which means that diameter of fibers was not statistically different in case of aligned and not-aligned fibers.

## 4. Conclusions

In this study, cylindrical nanofibrous structures with aligned and not- aligned fibers of PAN, PLGA, PCL and PVA were fabricated using conventional and novel collectors developed via electrospinning method for vascular graft applications. The obtained results showed that the average fiber diameter and porosity values of cylindrical nanofibrous structures with aligned fibers were lower than those with not- aligned fibers, whereas desired higher mechanical properties and suture retention properties were observed in the former case when compared to the latter case. It was also observed that burst pressure of cylindrical structures with aligned nanofibers was comparable to the grafts needed for vascular implants. Among all the polymers studied, aligned PLGA structures showed the best overall mechanical strength, burst pressure (350 ± 50 mmHg) and suture retention strength (27 N), being quite comparable to native blood vessels, which makes it a promising candidate for application as artificial blood vessels. 

## Figures and Tables

**Table 1:** Analysis of Variance (ANOVA) for different response variables.

(a) ANOVA for Fiber Orientation					
Parameter	DF	SS	MS	F	P
POLYMER	3	117.7	39.2	13.58	0.000 *
ALIGNMENT	1	39,627.0	39,627.0	13,721.95	0.000 *
Error	35	101.1	2.9		
Total	39	39,845.8			
**(b) ANOVA for Fiber Diameter**					
Parameter	DF	SS	MS	F	P
POLYMER	3	459,946	153,315	77.49	0.000 *
ALIGNMENT	1	21	21	0.01	0.918
Error	35	69,252	1979		
Total	39	529,219			
**(c) ANOVA for Tensile Strength**					
Parameter	DF	SS	MS	F	P
POLYMER	3	125.94	41.981	34.20	0.000 *
ALIGNMENT	1	395.64	395.641	322.34	0.000 *
Error	35	42.96	1.227		
Total	39	564.54			
**(d) ANOVA for Suture Retention Strength**					
Parameter	DF	SS	MS	F	P
POLYMER	3	902.50	300.83	1815.37	0.000 *
ALIGNMENT	1	1000.00	1000.00	6034.48	0.000 *
Error	35	5.80	0.17		
Total	39	1908.30			
**(e) ANOVA for Burst Pressure**					
Parameter	DF	SS	MS	F	P
POLYMER	3	21,700	7233	14.08	0.000 *
ALIGNMENT	1	883,278	883,278	1719.68	0.000 *
Error	35	17,977	514		
Total	39	922,955			

* Statistically significant; DF = degree of freedom; SS = sum of squared deviation; MS = Mean squared deviation; F = F-static; P = probability of null hypothesis.

## Data Availability

Not applicable.
